# Derelict Fishing Line Provides a Useful Proxy for Estimating Levels of Non-Compliance with No-Take Marine Reserves

**DOI:** 10.1371/journal.pone.0114395

**Published:** 2014-12-29

**Authors:** David H. Williamson, Daniela M. Ceccarelli, Richard D. Evans, Jos K. Hill, Garry R. Russ

**Affiliations:** 1 Australian Research Council Centre of Excellence for Coral Reef Studies, Townsville, Queensland 4811, Australia; 2 James Cook University, School of Marine and Tropical Biology, Townsville, Queensland 4811, Australia; 3 Department of Parks and Wildlife, 17 Dick Perry Ave., Kensington, Perth, Western Australia 6151, Australia; 4 Oceans Institute, School of Plant Biology, University of Western Australia, Crawley, WA 6009, Australia; 5 Olazul, 150 Post Street, Suite 342, San Francisco, CA, 94108, United States of America; Dauphin Island Sea Lab, United States of America

## Abstract

No-take marine reserves (NTMRs) are increasingly being established to conserve or restore biodiversity and to enhance the sustainability of fisheries. Although effectively designed and protected NTMR networks can yield conservation and fishery benefits, reserve effects often fail to manifest in systems where there are high levels of non-compliance by fishers (poaching). Obtaining reliable estimates of NTMR non-compliance can be expensive and logistically challenging, particularly in areas with limited or non-existent resources for conducting surveillance and enforcement. Here we assess the utility of density estimates and re-accumulation rates of derelict (lost and abandoned) fishing line as a proxy for fishing effort and NTMR non-compliance on fringing coral reefs in three island groups of the Great Barrier Reef Marine Park (GBRMP), Australia. Densities of derelict fishing line were consistently lower on reefs within old (>20 year) NTMRs than on non-NTMR reefs (significantly in the Palm and Whitsunday Islands), whereas line densities did not differ significantly between reefs in new NTMRs (5 years of protection) and non-NTMR reefs. A manipulative experiment in which derelict fishing lines were removed from a subset of the monitoring sites demonstrated that lines re-accumulated on NTMR reefs at approximately one third (32.4%) of the rate observed on non-NTMR reefs over a thirty-two month period. Although these inshore NTMRs have long been considered some of the best protected within the GBRMP, evidence presented here suggests that the level of non-compliance with NTMR regulations is higher than previously assumed.

## Introduction

Overharvesting and habitat degradation caused by destructive fishing methods are among the greatest threats to the health and productivity of marine ecosystems [1]–[3]. In addition to the direct ecological effects of fishing, lost or discarded (derelict) fishing gear continues to kill, injure or damage marine organisms and communities [4]–[9]. Almost all fisheries are subject to some level of gear loss or abandonment, mostly through entanglement with the benthos, severe weather and resource user conflicts [10]–[11]. Hook-and-line is a commonly used fishing method on coral reefs and rates of gear loss are often relatively high, particularly when fishing over highly complex reef slope habitats, with steep walls, overhangs, caves and high hard coral cover [6], [10], [12]. Although the direct impacts of hook and line fishing are generally lower than more habitat-destructive fishing methods [2], [13], derelict fishing line can be detrimental to the health of corals and other benthic invertebrates [5], [9], [14]. Furthermore, nylon fishing line is extremely persistent in the marine environment, potentially remaining intact for decades or even centuries [15]–[16]. Once entangled on coral reefs, derelict fishing lines eventually become overgrown by benthic invertebrates and ultimately they are embedded in the reef matrix [5].

Networks of no-take marine reserves (NTMRs) are increasingly being established to conserve or restore biodiversity and to enhance the persistence and sustainability of exploited fish populations [17]–[19]. Adequately protected NTMRs have been shown to effectively reduce habitat degradation, boost the density, size and biomass of fishery-targeted species and restore fish and benthic assemblages to more natural states [20]–[21]. Furthermore, several recent studies have demonstrated that NTMRs can provide both adult spillover and recruitment subsidies to surrounding fished areas, potentially enhancing the sustainability of exploited stocks and offsetting reductions in total fishable area [22]–[27].

Rapid increases in populations of targeted fish species have been recorded within some newly established NTMRs [28]–[29], however, significant increases in fish density, mean size and biomass can take many years to accrue and potentially decades to stabilize at carrying capacity [30]–[31]. Numerous factors can influence the performance of NTMR networks in meeting conservation or fishery management objectives including the pre-existing condition of benthic and fish communities within newly established NTMRs and surrounding fished areas, the size and spacing of reserves, the duration of protection, and the level of fisher compliance with reserve closures [32]–[36]. Non-compliance with NTMRs can limit or negate population recovery, and any accrued gains can be quickly eroded if compliance breaks down [34], [37]–[38].

Reliable estimates of the levels of NTMR non-compliance are often difficult and costly to obtain, but they are fundamental to determining the ecological impact of infringements, the effectiveness of surveillance operations and public awareness programs, and for gauging the capacity for NTMRs to meet stated objectives [33], [39]–[41]. Surveillance of fisher compliance with NTMRs in the Great Barrier Reef Marine Park (GBRMP) is conducted by several government agencies using a combination of vessel and aircraft patrols, and a satellite vessel monitoring system (VMS) for commercial trawl fishers [42]. Non-compliance of fishers with NTMRs in the GBRMP has long been recognized as a problem, and since 2004 hundreds of infringements by commercial and recreational fishers have been recorded annually [33], [42]–[44]. However, given the enormous size of the GBRMP, the remoteness of much of it, and the limited financial and logistical resources allocated to surveillance and enforcement, it is highly likely that the recorded infringements represent a small proportion of the total.

Several previous studies have used underwater visual surveys of derelict fishing gear to assess the fishing methods being applied in various areas, the ecological impacts of the gear itself, and the spatial distribution of fishing effort [6], [10], [12], [45]. However, monitoring of derelict fishing gear has not been previously used to estimate the levels of fishing effort within NTMRs relative to surrounding fished areas.

The key objectives of the present study were to:

1. Quantify densities of derelict fishing line and benthic community attributes on NTMR and non-reserve reefs in the Palm, Whitsunday and Keppel Island groups to provide insight into regional variation in fishing effort and NTMR non-compliance.
2. Measure the accumulation rates of derelict fishing line on NTMR and non-NTMR reefs in the Palm Islands and provide a direct estimate of NTMR non-compliance levels.

## Materials and Methods

### Study location and background

**Figure 1:**
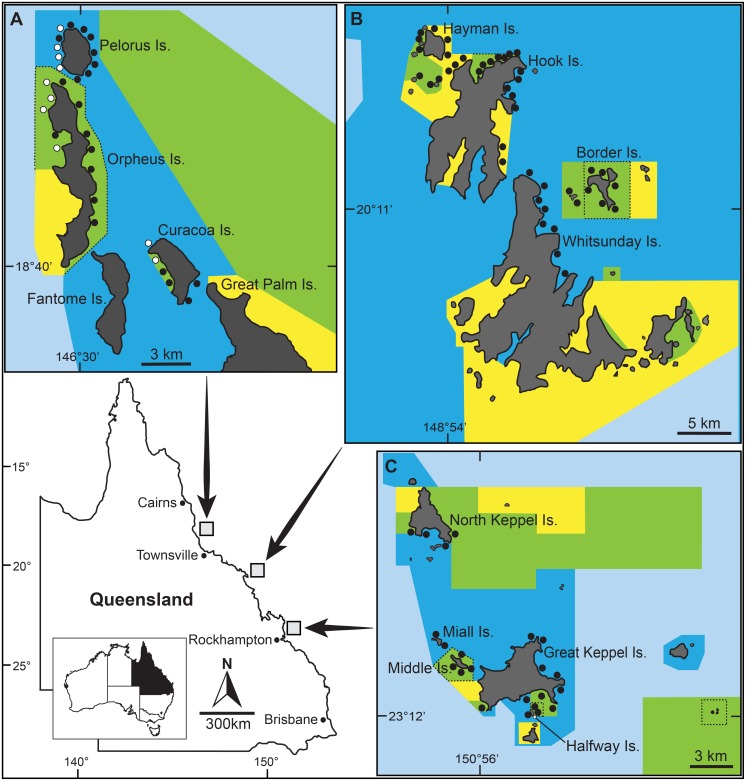
Study location map, showing the post-2004 management zoning for the Palm (A), Whitsunday (B) and Keppel (C) Island groups. Green shaded areas are no-take marine reserves (Marine National Park zones) that prohibit all fishing (NTMRs); Yellow shaded areas (Conservation Park zones) permit restricted recreational fishing; Dark blue shaded areas (Habitat Protection zones) are open to all forms of fishing except demersal trawling; Light blue shaded areas (General Use zones) are open to all forms of fishing. NTMRs that were established in 1987 are bordered by dashed lines, all other NTMRs were established in 2004. Circular markers indicate the approximate location of all (94) monitoring sites. The 10 sites in the Palm Island group at which derelict fishing lines were removed and line re-accumulation was quantified are labeled with white markers and site name codes.

This study was conducted on fringing coral reefs surrounding continental islands in the Palm, Whitsunday and Keppel Island groups, within the Great Barrier Reef Marine Park (GBRMP), Australia (Fig. 1). Apart from several tourist resorts, camping grounds and relatively small leasehold areas, the majority of the islands within these archipelagos are uninhabited national parks. Due to their outstanding natural beauty and ease of access from the mainland, these archipelagos are exposed to significant levels of tourism use and recreational fishing effort [42], [46]. Multiple-use management zoning plans, including networks of NTMRs, were first introduced to these island groups in 1987. An updated zoning plan was implemented within the entire GBRMP in July 2004 and several new NTMRs were established in the Palm, Whitsunday and Keppel Islands [47].

The experimental component of this study was conducted at Orpheus, Pelorus and Curacoa Islands in the Palm Island group (Fig. 1). The majority of the fringing reef surrounding Orpheus Island has been zoned as a NTMR since 1987. Challenger Bay on the western side of Curacoa Island was established as a NTMR in July 2004. The remainder of the fringing reef surrounding Curacoa Island and the entire reef surrounding Pelorus Island are open to hook and line fishing, spear fishing and collecting.

Great Palm Island is the largest island in the Palm Island group and it has a resident Aboriginal community of around 2500 people. Several islands within the Palm Island group, including Curacoa Island, and their surrounding waters and reefs are under Aboriginal tenure. In accordance with Australia’s Native Title Act (1993), traditional use of marine resources by Aboriginal traditional owners (including hunting, fishing and collecting) is permitted within the GBRMP, including within Marine National Park Zones (NTMRs) [47].

The recognized traditional owners of the Palm Island group are the Manbarra people, who have had an association with the area for thousands of years [48]. Great Palm Island was established as a penal settlement in 1918 and over the following decades, at least 1600 people from over 40 different Aboriginal tribes and language groups from across Queensland were forcibly removed from their traditional lands by the state government and resettled on the island. The majority of the current residents of Great Palm Island are the descendants of these displaced peoples, and collectively they are the Bwgcolman (Palm Island) people [48]. Despite not being true traditional owners, the Bwgcolman people clearly have a strong historical connection to Palm Island. The general approach of marine park surveillance officers has been to assume that all sighted indigenous fishers in the Palm Islands have legitimate fishing and hunting access to all areas, including NTMRs (GBRMPA pers. com.).

### Survey methods

Underwater visual census (UVC) was used to survey derelict fishing line, the benthic community and the structural complexity of the benthic habitat, at 30 sites in the Palm Islands, 42 sites in the Whitsunday Islands and 22 sites in the Keppel Islands during 2009. Within each island group, half of the sites (n = 47) were located on reefs within NTMRs and half (n = 47) were on reefs that are open to fishing (non-NTMR). A proportion of the NTMR sites in each island group were located within ‘old’ reserves (NTMR 1987) protected since 1987; while others were located within ‘new’ reserves (NTMR 2004) protected since 2004 (Table 1).

**Table 1:** The number of underwater visual census (UVC) sites surveyed within old no-take marine reserves (NTMR 1987), new no-take marine reserves (NTMR 2004) and non-NTMRs, in the Palm, Whitsunday and Keppel Island groups during 2009.

Island Group	Zone	No. UVC Sites
Palm	NTMR 1987	12
Palm	NTMR 2004	3
Palm	Non-NTMR	15
Whitsunday	NTMR 1987	12
Whitsunday	NTMR 2004	9
Whitsunday	Non-NTMR	21
Keppel	NTMR 1987	6
Keppel	NTMR 2004	5
Keppel	Non-NTMR	11

Surveys were conducted on reef slopes at depths between 4 m and 9 m depending on the reef slope topography at each site. Derelict fishing lines were surveyed on five replicate 50 m×6 m (300 m^2^) transects at each site. The total area surveyed for derelict lines at each site was 1500 m^2^. The benthic community was surveyed on the same transects using a standard line-intercept survey method every 1 m along each transect (50 points per transect). Hard corals were classified as live or dead and assigned into morphological categories (branching, digitate, plate, massive, foliose, encrusting). Other categories of benthos included soft coral, sponges, clams (*Tridacna* spp.), other invertebrates (such as ascidians and anemones), macro-algae, coral reef pavement, rock, rubble and sand. Reef structural complexity was estimated using a five-point scale for both reef slope angle and rugosity (Table 2). Five independent structural complexity estimates were made for each transect. Underwater visibility was recorded on each transect and ranged from 6 m to 12 m. Surveys did not proceed if the visibility was less than 5 m. All UVC were conducted on SCUBA by two observers (D. Williamson and R. Evans).

**Table 2:** Description of categories for reef slope and rugosity (habitat complexity), estimated visually for each underwater visual census (UVC) transect.

Category	Description of reef slope & rugosity categories
1	Reef slope 0–10°. Expanses of rubble and sand with some small scattered bommies
2	Reef slope <45°. Bommies dispersed amongst mostly rubble and sand
3	Reef slope ∼45°. Small rubble and sand patches amongst bommies and/or coral structure
4	Reef slope >45°. Complex coral structure, bommies, some small over-hangs, holes and caves
5	Reef slope ∼90°. High reef complexity, large over-hangs, holes, caves and bommies

### Removal of derelict fishing line and monitoring of line re-accumulation

Teams of volunteer divers from ReefCheck Australia carried out the removal of derelict fishing lines at ten of the monitoring sites in the Palm Islands during April 2007. Five of the cleaned sites were located on reefs within NTMRs and five were on non-NTMR reefs. Four of the cleaned NTMR sites were located on the leeward side of Orpheus Island (NTMR 1987) and one site was at Curacoa Island (NTMR 2004). Of the cleaned non-NTMR sites, four were located on the leeward side of Pelorus Island and one was at Curacoa Island (Fig. 1). The ten cleaned sites were randomly selected from a pool of eighteen sites that were in sheltered (leeward) locations and were identified as being readily accessible by fishers during calm to moderate weather conditions (<15 knots of wind).

At each site, between 4 and 6 divers worked in teams to remove all sighted derelict fishing lines and tackle (hooks, sinkers, wire leaders etc.) on reef slopes between 4 m and 12 m depth, for 200 m in both directions from a central GPS waypoint marking the site position. Transect tapes were deployed to measure the distance covered and increase the search efficiency so that very few, if any, derelict fishing lines remained after cleanup operations were completed. Approximately 4 hours of dive time was spent on each site by the cleanup team (16–24 diver-hours per site). Derelict lines that were overgrown with hard coral or encrusting sponges, or partially embedded in the reef matrix were cut, and all of the exposed (visible) sections of line were removed. All collected fishing lines were recorded on data sheets before being placed in catch bags and taken back to the boat for later disposal.

Baseline UVC surveys of derelict fishing lines were conducted at each of the ten cleaned sites immediately prior to the removal of fishing lines in April 2007 and UVC surveys were subsequently repeated in December 2009, providing a 32 month period for fishing lines to re-accumulate on the reefs. The 2007 and 2009 UVC surveys on the 10 cleaned sites used the same methodology described in the *survey methods* section above.

We assumed that all of the fishing lines recorded and collected were lost at the same location in which they were found. The authors recognize that the removal of derelict fishing line from ten of the thirty sites in the Palm Islands prior to the 2009 surveys would have reduced the fishing line density estimates for the Palm Islands. However the magnitude of this effect was balanced between old and new NTMRs and non-NTMRs, as one-third of the monitoring sites in each zone were cleaned. Fieldwork was conducted under Great Barrier Reef Marine Park research permit number G06/19976.1.

### Data treatment and analyses

#### Fishing line density, benthic cover and community composition

Two-factor analysis of variance (ANOVA) was used to test for differences in fishing line density, live hard coral cover (LHCC) and habitat structural complexity (SC) between island groups and between management zones within each island group, using 2009 UVC data. ‘Island group’ (3 levels – Palm, Whitsunday, Keppel) and ‘zone’ (3 levels – NTMR 1987, NTMR 2004, non-NTMR) were treated as fixed factors. For this regional-scale analysis, transect counts of derelict fishing lines were summed for each site, while LHCC and SC estimates were averaged for each site. Analyses were conducted using the site-level summed or mean values as the replicates. To comply with ANOVA assumptions, site-level density estimates of derelict fishing line were log (x +1) transformed, while LHCC and SC estimates were log (x) transformed.

Two-factor ANOVA was also applied to counts of derelict fishing line on the 10 sites that were cleaned in the Palm Islands with ‘zone’ (2 levels – NTMR and non-NTMR) and ‘year’ (2 levels–2007 and 2009) as fixed factors. Single-factor ANOVA was used to compare the benthic attributes (SC and LHCC) of the 10 cleaned sites in 2009 with ‘zone’ (2 levels – NTMR and non-NTMR) treated as the fixed factor. Analyses of fishing line density, SC and LHCC at the 10 cleaned sites were conducted using site-level data as the replicates. Raw fishing line counts were log (x +1) transformed, while LHCC and SC estimates were log (x) transformed to comply with ANOVA assumptions.

We also used non-metric multi-dimensional scaling (nMDS) analysis to partition differences in benthic community composition between regions (island groups) and between NTMR and non-NTMR reefs within each island group [49]. Differences in benthic community composition were also assessed for the 10 cleaned sites in the Palm Island group using nMDS. All benthic community data were square root – arcsine transformed prior to nMDS.

A non-metric, 1-way, pairwise analysis of similarity (ANOSIM) among the groups, and a SIMPER analysis [50] was also conducted to determine the individual benthic categories that accounted for the similarities and differences among regions and between management zones within regions. To determine the relative contribution of the benthic categories to the final nMDS solution, each variable was projected onto the ordination space. Vectors were calculated using the partial regression coefficients of the original variables within the two dimensions of the nMDS, and the lengths of the vectors were set proportional to the squared multiple correlation coefficient.

### Estimates of the accumulation rate of derelict fishing line

At each site the UVC survey area (1500 m^2^) was smaller than the total area cleaned of lines. Furthermore, variation in reef slope habitat characteristics among sites may have influenced the UVC sighting efficiency rate of derelict fishing lines. Therefore, to estimate the accumulation rate of derelict fishing lines for sites that had been cleaned, it was first necessary to calculate the site-specific UVC line sighting efficiency rates. Total UVC line counts for each site in the April 2007 baseline survey were compared with the total number of lines removed from each site. The line sighting efficiency factor was then calculated for each site using equation 1 below.
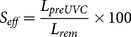
(1)


Where;

*S_eff_* = UVC line sighting efficiency

*L_preUVC_* = Pre-cleanup UVC total line count

*L_rem_* = Total number of lines removed

The UVC line sighting efficiency rate for each of the 10 cleaned sites ranged from 21% to 100% with a mean of 45.7% (±7.3% SE). Line accumulation rates were thus calculated independently for each site. To estimate the total number of lines that had re-accumulated at each site, total counts of derelict fishing line from the 2009 post-cleanup UVC survey were scaled by the site-specific line sighting efficiency rate according to equation 2 below.
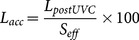
(2)


Where;

*L_acc_* = Total number of lines accumulated

*L_postUVC_* = Post-cleanup UVC total line count

*S_eff_* = UVC line sighting efficiency

The resulting total fishing line accumulation values were subsequently divided by the number of months between the site cleanup and the post-cleanup UVC survey [32] to provide a site-specific estimate of the monthly line accumulation rate. Single-factor ANOVA was used to test for differences between zones (NTMR and non-NTMR) in both the total number of derelict fishing lines removed and the line accumulation rates.

## Results

### Regional patterns of derelict fishing line density

**Figure 2:**
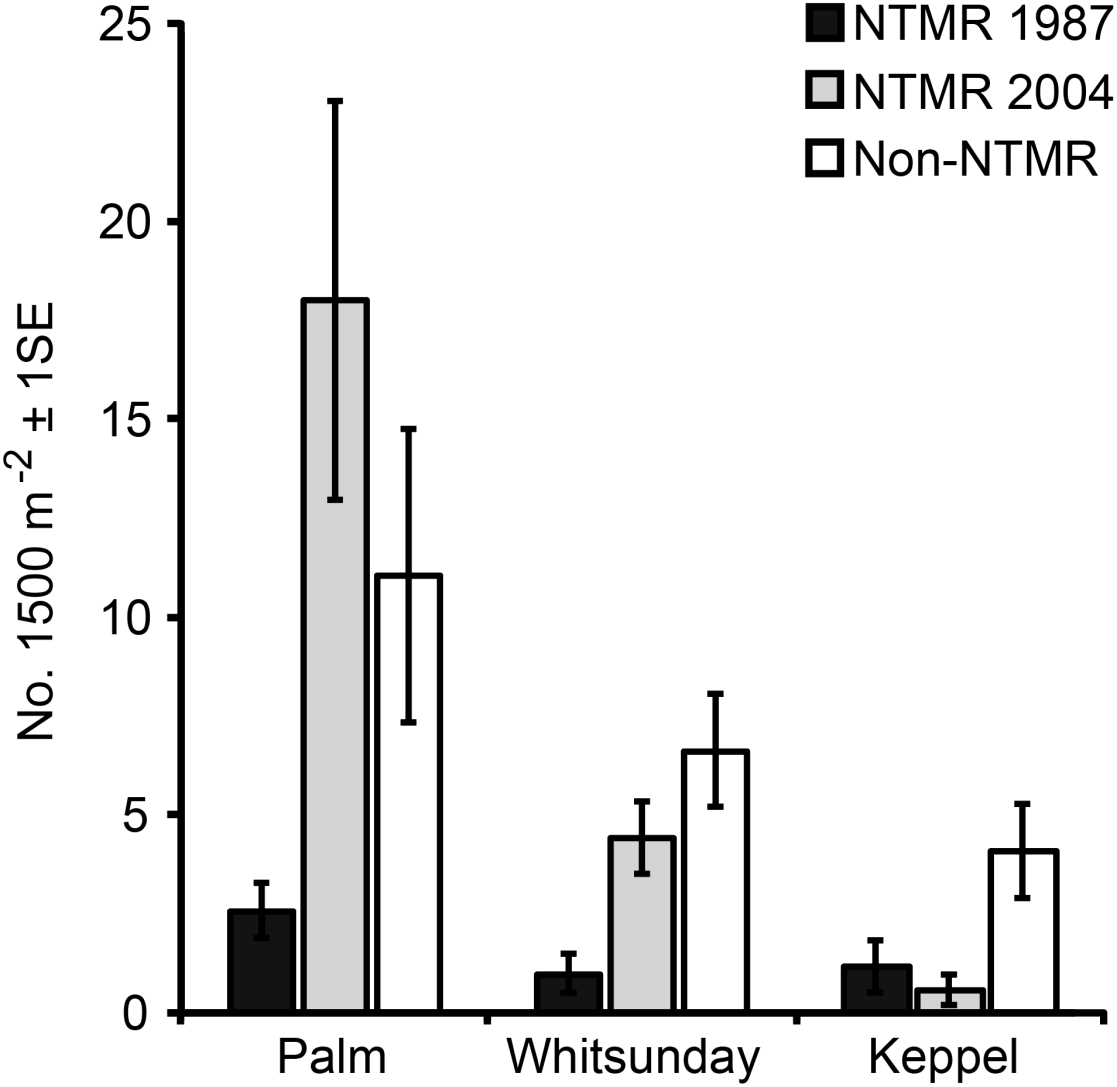
Mean (±1SE) density of derelict fishing lines on reefs within NTMRs and non-NTMR zones of the Palm, Whitsunday and Keppel Island groups during 2009.

During the 2009 UVC surveys of reefs in the Palm, Whitsunday and Keppel Island groups, derelict fishing lines were recorded within old (1987) NTMRs, new (2004) NTMRs and in non-NTMR zones that are legally open to fishing. Significant differences in overall mean fishing line density were detected between the three study regions (ANOVA, F_2,85_ = 9.01, *p*<0.001) (Fig. 2). The mean line density on reefs in the Palm Islands was nearly twice as high as in the Whitsunday Islands, and more than three times higher than in the Keppel Islands. These observed regional differences in line density were significant between the Palm and Keppel Islands (Tukey HSD, *p*<0.01), however the differences between the Palm and Whitsunday Islands and between the Whitsunday and Keppel Islands were not significant.

Fishing line densities varied significantly between NTMR and non-NTMR reefs in the Palm and Whitsunday Islands, but not in the Keppel Islands (ANOVA, F_4,85_ = 8.62, *p*<0.001) (Fig. 2). In the Palm Islands, line densities were significantly higher in new NTMRs than in old NTMRs (Tukey HSD, *p*<0.05), but there was no significant difference in the density of lines between non-NTMRs and both old and new NTMRs (Fig. 2). In the Whitsunday Islands, line densities were significantly higher in non-NTMRs than in old NTMRs (Tukey HSD, *p*<0.01), but there was no significant difference in line densities between non-NTMRs and new NTMRs, or between old and new NTMRs (Fig. 2). Although the mean density of fishing lines was higher in non-NTMRs than in both old and new NTMRs in the Keppel Islands, these differences were not significant (Fig. 2).

### Reef habitat complexity, hard coral cover and benthic community composition

**Figure 3:**
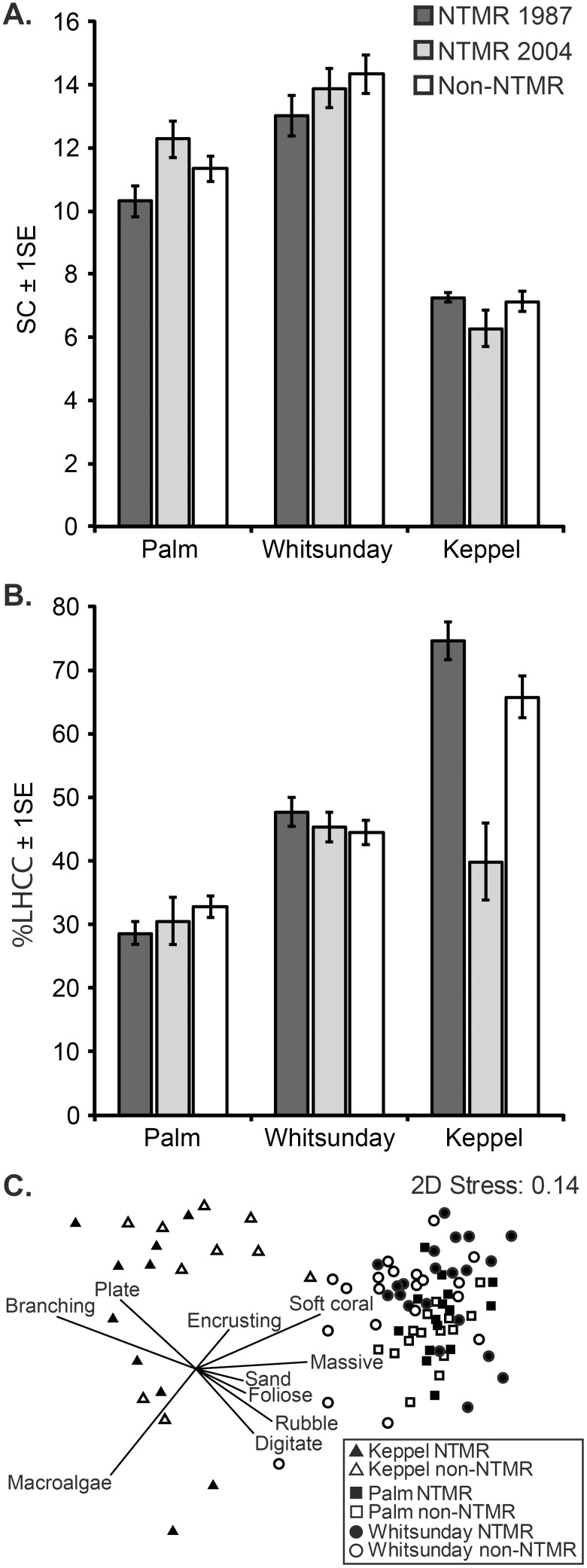
A. Mean (±1SE) habitat structural complexity index (SC), B. Mean (±1SE) percent live hard coral cover (%LHCC), C. Non-metric Multidimensional Scaling (nMDS) biplot on the Bray-Curtis similarity matrix of log (x+1) transformed benthic community data (coral morphological groupings, rubble and sand) on reefs within NTMRs and non-NTMR zones of the Palm, Whitsunday and Keppel Island groups during 2009.

Reef habitat structural complexity (SC) was significantly higher in the Whitsunday Islands than in the Palm and Keppel Islands (ANOVA, F_2,85_ = 27.41, *p*<0.001) (Fig. 3a). However, there were no significant differences in SC between NTMR and non-NTMR zones within each of the three island groups. Live hard coral cover (LHCC) was significantly lower in the Palm Islands than in the Whitsunday and Keppel Islands (ANOVA, F_2,85_ = 9.50, *p*<0.001) (Fig. 3b). In the Palm and Whitsunday Islands there were no significant differences in LHCC between NTMR and non-NTMR zones, while in the Keppel Islands, LHCC was significantly lower in new NTMRs than in both old NTMRs and non-NTMRs (Tukey HSD, *p*<0.05) (Fig. 3b).

Benthic community composition also varied significantly among regions (ANOSIM, Global R = 0.572, *p*<0.01) with massive corals and soft corals accounting for a higher proportion of the reef benthos in the Palm and Whitsunday Islands and branching and plate corals dominating reefs in the Keppel Islands (Fig. 3c, Table 3). Benthic community composition was similar between NTMR reefs and non-NTMR reefs within each island group with average dissimilarities of 27.5% (Palm Islands), 34.3% (Whitsundays Islands) and 41.3% (Keppel Islands) (Table 3 and Table S1 in S1 File).

**Table 3:** Results of SIMPER analysis on the percent cover of benthic categories (square root – arcsine transformed, Bray-Curtis similarity matrix), testing dissimilarity between regions (Palm, Whitsunday, Keppel) and between NTMR and Non-NTMR zones within regions during 2009.

*Benthic category*	*Contribution %*	*Cumulative %*	*Benthic category*	*Contribution %*	*Cumulative %*
*Groups: Palm & Whitsunday* - Average dissimilarity = 33.6%			*Groups: Palm NTMR & Non-NTMR* - Average dissimilarity = 27.5%		
Rubble	16.8	16.8	Soft coral	15.4	15.4
Soft coral	12.9	29.7	Rubble	12.8	28.2
Massive	12.8	42.5	Digitate	12.4	40.6
Digitate	10.1	52.6	Encrusting	11.0	51.7
			Foliose	10.7	62.4
			Sand	10.5	72.9
*Groups: Keppel & Palm* - Average dissimilarity = 68.5%			*Groups: Whitsunday NTMR & Non-NTMR* - Average dissimilarity = 34.3%		
Branching	25.8	25.8	Massive	13.9	13.9
Soft coral	15.1	40.9	Soft coral	13.9	27.9
Rubble	12.0	53.0	Branching	11.6	39.5
Macroalgae	12.0	65.0	Encrusting	11.1	50.6
*Groups: Keppel & Whitsunday* - Average dissimilarity = 65.6%			*Groups: Keppel NTMR & Non-NTMR* - Average dissimilarity = 41.3%		
Branching	25.2	25.2	Macroalgae	27.4	27.4
Soft coral	17.1	42.3	Branching	21.1	48.5
Massive	14.7	57.0	Plate	10.2	58.7
Macroalgae	13.7	70.7			

Only benthic categories that accounted for at least 10% of the dissimilarity are included.

**Figure 4:**
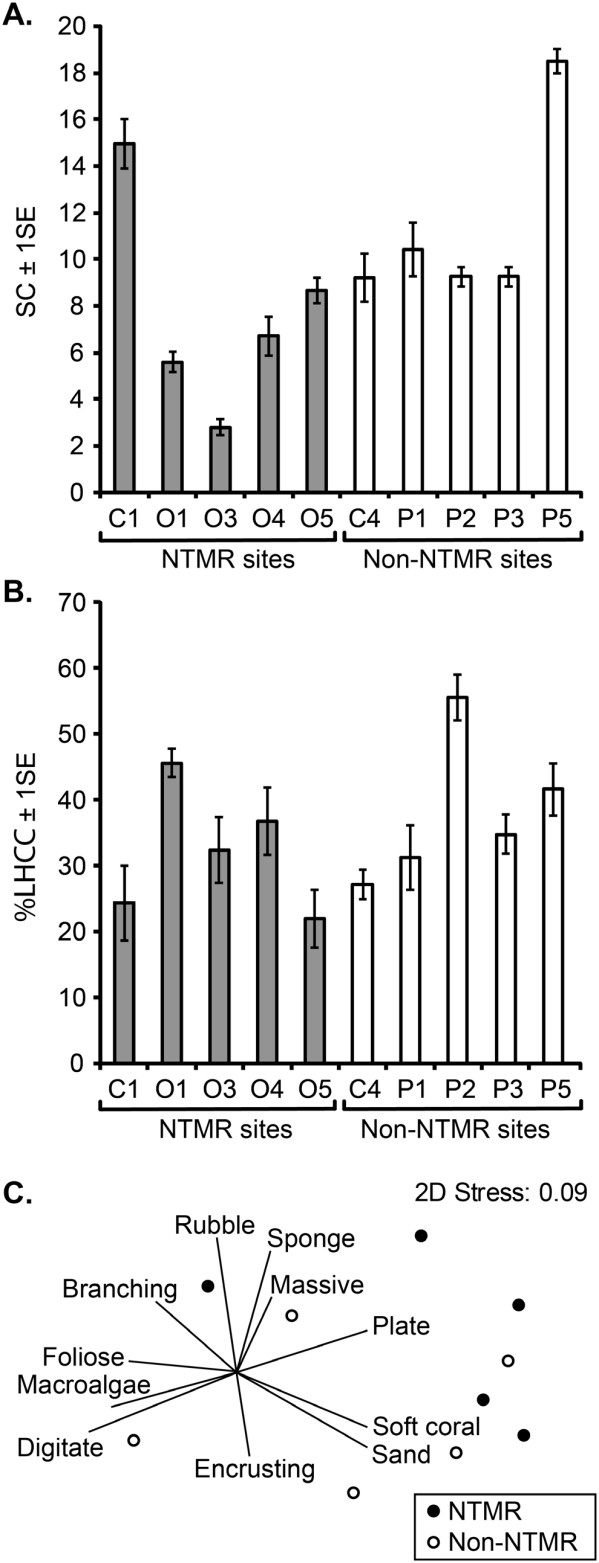
Mean (±1SE) habitat structural complexity index (SC), B. Mean (±1SE) percent live hard coral cover (%LHCC), C. Non-metric Multidimensional Scaling (nMDS) biplot on the Bray-Curtis similarity matrix of log (x+1) transformed benthic community data (coral morphological groupings, rubble and sand) at 5 cleaned NTMR sites and 5 cleaned non-NTMR sites in the Palm Island group during 2009.

Habitat structural complexity (SC) was significantly higher at the 5 cleaned non-NTMR sites than at the 5 cleaned NTMR sites in the Palm Islands (ANOVA, F_1,8_ = 12.48, *p*<0.01) (Fig. 4a). Live hard coral cover did not vary significantly between the cleaned NTMR and non-NTMR sites (Fig. 4b). Although some differences in benthic community composition were apparent among the 10 cleaned sites in the Palm Islands (Fig. 4c), the average dissimilarity between NTMR and non-NTMR sites was only 26.3% (Table 4 and Table S2 in S1 File). The overall variation in benthic community composition between NTMR and non-NTMR sites was not significant (ANOSIM, Global R = 0.012, *p*>0.05).

**Table 4:** Results of SIMPER analysis on the percent cover of benthic categories (square root – arcsine transformed, Bray-Curtis similarity matrix), testing dissimilarity between 5 cleaned NTMR sites and 5 cleaned non-NTMR sites in the Palm Island group during 2009.

*Benthic category*	*Contribution %*	*Cumulative %*
*Groups: Palm NTMR & Non-NTMR* - Average dissimilarity = 26.3%		
Digitate	15.6	15.6
Soft coral	12.1	28.0
Foliose	12.0	40.0
Rubble	11.7	51.7
Sand	10.3	62.0
Encrusting	10.1	72.1

Only benthic categories that accounted for at least 10% of the dissimilarity are included.

### Removal and re-accumulation of derelict fishing line in the Palm Islands

**Figure 5:**
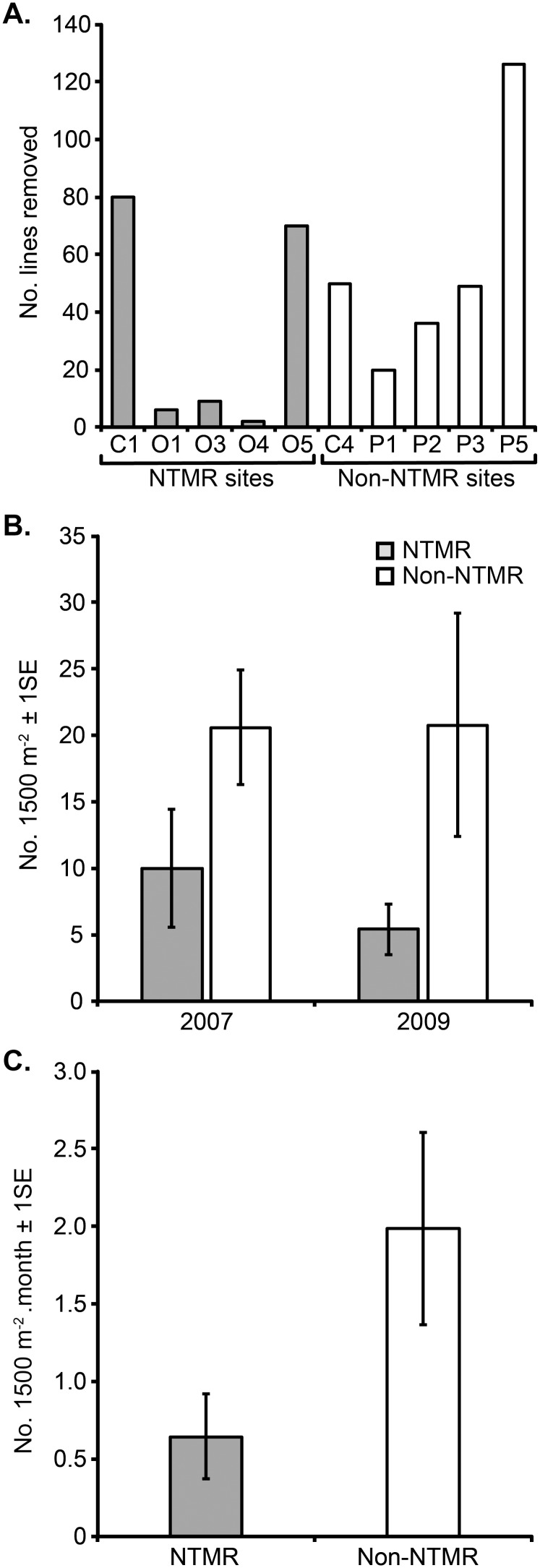
A. Total number of derelict fishing lines removed from 5 NTMR and 5 non-NTMR monitoring sites in the Palm Island group during 2007. **B.** Mean (±1SE) density of derelict fishing lines in the same 5 NTMR and 5 non-NTMR sites prior to line removal in 2007 and approximately 32 months after line removal in 2009. **C**. Mean (±1SE) accumulation rate of derelict fishing lines (number of lines per month) at the same 5 NTMR and 5 non-NTMR sites between 2007 and 2009.

A total of 281 derelict fishing lines were removed from the five non-NTMR sites, and 167 lines were removed from the five NTMR sites (Fig. 5a). There was no significant difference in the mean number of lines removed from non-NTMR and NTMR zones (ANOVA, F_1,8_ = 0.83, *p*>0.05). Two of the three sites with the greatest number of lines removed were within NTMRs. One of those sites (O5) had been protected since 1987 (∼20 years at the time of line removal), while the other (C1) had been protected since 2004 (∼3 years at the time of line removal) (Fig. 5a). Almost 90% of the lines removed from NTMR sites (150 out of 167) were sourced from site O5 and site C1. Conversely, the three sites with the lowest number of lines removed (O1, O3, O4) were all within the old NTMR at Orpheus Island (Fig. 5a).

In April 2007, prior to the commencement of clean-up operations, the mean density of derelict fishing lines was higher, but not significantly so, in the five non-NTMR sites than in the five NTMR sites (Fig. 5b). In December 2009, thirty-two months after the ten sites were cleaned, the mean density was again higher, but not significantly, in non-NTMRs than in NTMRs (Fig. 5b). Between 2007 and 2009, fishing line re-accumulated to varying degrees at all sites that had been cleaned in 2007. The overall mean line accumulation rate within NTMRs was approximately one third (32.4%) of the accumulation rate within non-NTMRs (Fig. 5c). The difference in the mean accumulation rate of derelict fishing lines between NTMRs and non-NTMRs was marginally non-significant (ANOVA, F_1,8_ = 3.89, *p* = 0.084).

## Discussion

Densities of derelict fishing line, as estimated from UVC, provided useful insight into the regional differences in the distribution and intensity of fishing effort on fringing coral reefs of the GBRMP. However, it was evident that regional scale differences in reef habitat complexity, coral cover and benthic community composition may influence the loss rates of hook and line fishing gear, and these factors must be considered in the interpretation of the results. In the Palm and Whitsunday Islands, the lowest line densities were recorded on reefs within old NTMRs that had been closed to fishing for over twenty years. Furthermore, there were no significant differences in line density between non-NTMR reefs and new NTMR reefs with only 5 years of protection, in any of the island groups. Given that fishing line is extremely persistent in the marine environment, and that entangled lines can remain in place indefinitely, it is probable that a proportion of the fishing lines recorded on reefs within both old and new NTMRs were present on those reefs prior to reserve establishment.

Like most other marine debris, lost fishing line quickly becomes overgrown [fouled] with algae and other sessile organisms, particularly in tropical waters [6]. Although some un-fouled and thus recently lost, fishing lines were recorded in the surveys, the vast majority (>99%) had clearly been on the reefs for months to years, as they were either fouled or partially embedded in the reef matrix. It is therefore unlikely that the line densities observed within non-NTMRs and NTMRs in 2009 provided an accurate indication of the levels of NTMR non-compliance on these reefs. In order to effectively estimate non-compliance levels, it was necessary to remove all lines from a subset of the monitoring sites and measure the re-accumulation rates in both NTMR and non-NTMR sites. The findings from the experimental component of this study demonstrate that there was a significant level of non-compliance occurring within the NTMRs at Orpheus and Curacoa Islands in the Palm Island group.

Between 2007 and 2009, fishing lines accumulated on reefs within NTMRs at approximately one third the rate of that on non-NTMR reefs and there was no significant difference in the line accumulation rate between zones. Although live hard coral cover and benthic community composition were similar between the five cleaned non-NTMR sites and the five cleaned NTMR sites, habitat structural complexity was significantly higher at the non-NTMR sites. It may therefore be expected that rates of fishing gear loss would have tended to be higher in the non-NTMR sites. These findings suggest that between 2007 and 2009, NTMR reefs in the Palm Island group were subject to approximately 30% of the fishing effort that had been applied to non-NTMR reefs. Furthermore, this may be a conservative estimate due to the higher habitat structural complexity of the non-NTMR sites.

Two of the cleaned NTMR sites, O5 at the northwestern corner of Orpheus Island and C1 at Curacoa Island, were clear hotspots for illegal fishing. This pattern was evident in both the total number of lines removed from each site and the site-specific line accumulation rates. Of the five cleaned NTMR sites, both O5 and C1 were located closer to the boundaries of the reserves and were slightly more ‘out of sight’ than the other cleaned NTMR sites (O1, O3, O4) that were located on the leeward (western) side of Orpheus Island, in relatively close proximity to the Orpheus Island Research station and the Orpheus Island Resort. It is likely that ‘passive surveillance’ from both the research station and the resort may have had a significant influence on the locations in which fishers were choosing to fish illegally [44].

The observed re-accumulation of fishing lines suggest that there was a substantial amount of fishing occurring on NTMR reefs in Palm Islands between 2007 and 2009. It must be noted however, that we did not quantify the relative proportions of accumulated lines attributable to illegal fishing by recreational fishers and permitted traditional fishing by members of the Palm Island aboriginal community. The authors’ personal observations suggest that fishers from the Palm Island community predominantly fish the reefs surrounding Great Palm, Curacoa and Fantome Islands and rarely, if ever, fish the reefs within the NTMR on the leeward side of Orpheus Island. We therefore assume that only the two cleaned sites at Curacoa Island, C1 (NTMR) and C4 (non-NTMR) were subject to significant levels of fishing effort by fishers from the Palm Island community.

Although the broad scale (2009) survey data from the Palm, Whitsunday and Keppel Islands did not provide reliable estimates of NTMR non-compliance, it did provide a valuable regional-scale comparison of fishing effort, reef habitat attributes, and the line accumulation potential of different reef types. Significant regional differences in mean line density were detected, with the highest densities recorded on reefs in the Palm Island group and lower densities on reefs in the Whitsunday and Keppel Island groups. Regional differences in fishing effort may partly account for this result, however differences in reef structural complexity and benthic community composition are likely to have also accounted for a proportion of this variability.

In the present study, densities of fishing lines were higher on the more structurally complex reefs of the Palm and Whitsunday Islands than on the lower complexity reefs in the Keppel Islands. Although hard coral cover was highest in the Keppel Islands, the majority of the reefs were dominated by relatively fragile branching Acroporid corals, while reefs in the Palm and Whitsunday Islands had a higher cover of coral species with massive and digitate morphologies [51]. Fishers generally find it easier to retrieve entangled fishing gear from reefs in the Keppel Islands as branching corals are relatively easily broken or detached from the substrate. This may largely account for the significantly lower fishing line densities on reefs in the Keppel Islands. Conversely, reefs in the Whitsunday Islands had significantly higher structural complexity than reefs in the Palm Islands, yet they had significantly lower densities of derelict fishing line. This finding suggests that there were higher overall levels of fishing effort per unit area of reef in the Palm Islands than in the Whitsunday Islands.

Although some empirical data and a wealth of anecdotal information are available that suggests that non-compliance within NTMRs is widespread within the GBRMP [44], [52], it has been assumed that compliance levels are relatively high on inshore GBR reefs in comparison to more remote areas of the marine park [42], [44], [53]–[54]. The results of this study indicate that there is a surprisingly high level of fishing occurring on reefs within NTMRs in the Palm Islands and it is also apparent that there is some level of non-compliance in the Whitsunday and Keppel Islands. The findings suggest that NTMR non-compliance rates within the GBRMP may be significantly higher than previously assumed.

A number of previous studies that have assessed the ecological effects of NTMRs within the GBRMP have found significant benefits for key fishery target species such as coral trout (*Plectropomus* spp.), however the magnitude of the effects have often been spatially and temporally inconsistent [29], [51], [53], [55]–[58]. Generally, NTMR effects on the density, size and biomass of targeted fish species have been stronger and more consistent on inshore GBR reefs than on offshore reefs [29]. Variability in the magnitude of NTMR effects may be driven by a range of factors including the cross-shelf and latitudinal position of reefs their proximity to centers of human population and other reefs, the disturbance history of individual reefs and reef clusters, the quality and availability of habitats, oceanographic patterns, and the supply of larvae from upstream sources [42], [51]. However, given the findings presented here and those of several previous studies, it is evident that fisher non-compliance with NTMRs may also be an important contributing factor.

Although this study focused on the GBRMP, the findings have global relevance. The removal of derelict fishing gear and monitoring of gear re-accumulation has clear utility for independently quantifying the spatial distribution of fishing effort and the levels of non-compliance within NTMRs. Such data may prove highly relevant to assessments of the ecological effects of NTMRs as well as for informing community education programs and improving the effectiveness of marine park surveillance patrols.

## Supporting Information

S1 File **Contains Tables S1 and S2.**
(DOCX)
