# Supplementary material for: Derelict Fishing Line Provides a Useful Proxy for Estimating Levels of Non-Compliance with No-Take Marine Reserves
Source: PLoS One. 2014 Dec 29;9(12):e114395. doi: 10.1371/journal.pone.0114395 (PMC4278742; doi:10.1371/journal.pone.0114395)
Supplement: S1 File — Contains Tables S1 and S2. (DOCX) [file pone.0114395.s001.docx]

**Supporting Information**

**Table S1:** Mean (± 1SE) percent cover values for benthic categories in the three study regions (Palm, Whitsunday, Keppel) and between NTMR and Non-NTMR zones within regions during 2009. Only benthic categories that accounted for at least 10% of the dissimilarity identified by SIMPER analyses are included.

| *Benthic category* | *% Cover* | *% Cover* |  | *Benthic category* | *% Cover* | *% Cover* |
| --- | --- | --- | --- | --- | --- | --- |
| *Groups: Palm & Whitsunday* | | |  | *Groups: Palm NTMR & Non-NTMR* | | |
|  | Palm | Whitsunday |  |  | NTMR | Non-NTMR |
| Rubble | 21.1 (± 1.1) | 7.3 (± 0.5) |  | Soft coral | 25.9 (± 1.7) | 20.2 (± 1.6) |
| Soft coral | 23.1 (± 1.2) | 26.0 (± 1.1) |  | Rubble | 21.2 (± 1.5) | 20.9 (± 1.5) |
| Massive | 10.5 (± 0.7) | 19.2 (± 1.1) |  | Digitate | 5.5 (± 1.2) | 7.1 (± 1.1) |
| Digitate | 6.3 (± 0.8) | 6.5 (± 0.9) |  | Encrusting | 4.4 (± 0.6) | 6.2 (± 0.7) |
|  |  |  |  | Foliose | 4.0 (± 0.7) | 4.1 (± 0.7) |
|  |  |  |  | Sand | 5.1 (± 0.8) | 3.7 (± 0.7) |
|  |  |  |  |  |  |  |
|  |  |  |  |  |  |  |
| *Groups: Keppel & Palm* | | |  | *Groups: Whitsunday NTMR & Non-NTMR* | | |
|  | Keppel | Palm |  |  | NTMR | Non-NTMR |
| Branching | 57.5 (± 3.0) | 3.2 (± 0.3) |  | Massive | 24.9 (± 1.8) | 13.5 (± 1.1) |
| Soft coral | 2.1 (± 0.7) | 23.1 (± 1.2) |  | Soft coral | 29.4 (± 1.7) | 22.6 (± 1.4) |
| Rubble | 3.6 (± 0.6) | 21.1 (± 1.1) |  | Branching | 5.9 (± 0.8) | 8.7 (± 1.2) |
| Macroalgae | 19.8 (± 3.0) | 0.8 (± 0.2) |  | Encrusting | 2.6 (± 0.3) | 8.0 (± 0.8) |
|  |  |  |  |  |  |  |
|  |  |  |  |  |  |  |
| *Groups: Keppel & Whitsunday* | | |  | *Groups: Keppel NTMR & Non-NTMR* | | |
|  | Keppel | Whitsunday |  |  | NTMR | Non-NTMR |
| Branching | 57.7 (± 3.0) | 7.3 (± 0.7) |  | Macroalgae | 27.5 (± 4.7) | 12.1 (± 3.3) |
| Soft coral | 2.1 (± 0.7) | 26.0 (± 1.1) |  | Branching | 57.6 (± 4.8) | 57.4 (± 3.7) |
| Massive | 0.9 (± 0.2) | 19.2 (± 1.1) |  | Plate | 4.6 (± 1.0) | 9.7 (± 1.4) |
| Macroalgae | 19.8 (± 3.0) | 3.0 (± 0.8) |  |  |  |  |
|  |  |  |  |  |  |  |

**Table S2:** Mean (± 1SE) percent cover values for benthic categories at the 5 cleaned NTMR sites and 5 cleaned non-NTMR sites in the Palm Island group during 2009. Only benthic categories that accounted for at least 10% of the dissimilarity identified by SIMPER analyses are included.

| ***Benthic category*** | ***% Cover NTMR*** | ***% Cover Non-NTMR*** |
| --- | --- | --- |
| *Groups: Palm NTMR & Non-NTMR* | | |
| Digitate | 7.6 (± 1.9) | 13.6 (± 2.5) |
| Soft coral | 19.2 (± 2.5) | 18.7 (± 2.1) |
| Foliose | 1.4 (± 0.4) | 6.5 (± 1.8) |
| Rubble | 25.0 (± 2.4) | 20.5 (± 2.5) |
| Sand | 5.6 (± 1.6) | 3.5 (± 0.8) |
| Encrusting | 1.2 (± 0.3) | 3.9 (± 0.8) |
|  |  |  |
|  |  |  |
